# Determination of Cut Point in the Age of Colorectal Cancer Diagnosis Using a Survival Cure Model

**DOI:** 10.31557/APJCP.2019.20.9.2819

**Published:** 2019

**Authors:** Mahbubeh Abdollahi, Nayereh Kasiri, Mohamad Amin Pourhoseingholi, Ahmad Reza Baghestani, Habibollah Esmaily

**Affiliations:** 1 *Department of Public Health, School of Health,*; 2 *Health Sciences Research Center, Torbat Heydariyeh University of Medical Sciences, Torbat Heydariyeh,*; 3 *Gastroenterology and Liver Diseases Research Center, *; 4 *Department of Biostatistics, Faculty of Paramedical Sciences, Shahid Beheshti University of Medical Sciences, Tehran, *; 5 *Department of Biostatistics, Faculty of Health, Mashhad University of Medical Sciences, Mashhad, Iran. *

**Keywords:** Colorectal Cancer, survival analysis, survival cure model, cut point

## Abstract

**Background and Objectives:**

Colorectal Cancer (CRC) is the fourth cancer-related cause of death worldwide. CRC is a multi-stage cancer, which is curable during the early stages of the disease. Therefore, determining the time of cut-point existence could improve treatment planning and help directly allocate resources. This study aims to determine the cut point in the age of CRC diagnosis.

**Methods:**

This study, covering the course 1985 to 2012, consisted of 345 colorectal cancer patients registered in Taleghani Hospital, Tehran, Iran and followed up to 2013. The cut-point in the age of CRC diagnosis was obtained using a mixture cure model. The data were analyzed using SPSS and R, V. 20 and 2.15.0, respectively.

**Results:**

The results showed that the cut point in the age of CRC diagnosis was 50 years. Based on our estimation, 65% of the patients diagnosed with CRC at or younger than 50 were cured, while 31% of them diagnosed older than 50 were cured, and the younger group had a better survival over the older group.

**Conclusion:**

Since access to a cut-point and analysis of created prognostic groups are important in screening and treatment planning, our results suggested that it is better to estimate the cut-point in the age of curable cancers in early stages via survival cure models, and the cure rate would increase by CRC timely screening.

## Introduction

Cancer is a worldwide health problem and the second cause of human deaths in developed countries (Hanahan and Weinberg, 2000). Since cancer is the second leading cause of human death, (Hanahan and Weinberg, 2000) and plays an important role in health care, the advancement in the field of oncology in medical science is a main goal of health care programs (Goodman et al., 2006). Colorectal Cancer (CRC) is the third most common tumor and the fourth death leads to cancer worldwide (Li et al., 2009). CRC is a multistage cancer (Markowitz and Bertagnolli, 2009) where malignant cancer cells are formed in colon tissue (Garland and Garland, 1980). In Iran, incidence of this cancer has also increased in recent decades. Compared to Western countries, the CRC incidence is lower in Iran. It is reported as the fifth cancer in men and third in women (Moghimi-Dehkordi et al., 2008). Numerous factors contribute to the CRC development and metastasis such as age, diet, family history, smoking, inactivity, alcohol consumption, and obesity (Garland and Garland, 1980). 

Cancer cells inside colon walls can be treated with surgery in the first and second stages. If the disease is not treated at this stage, the cancer cells metastasize to nearby lymph nodes (third stage). In this stage, it is possible to cure through surgery and chemotherapy. In the fourth stage, cancer cells metastasize to more remote organs. Although the disease is usually not curable in this stage, chemotherapy may increase longevity (Markowitz and Bertagnolli, 2009).

In oncology studies categorizing a quantitative prognostic variable improves planning for treatment. Homogeneous prognostic groups are created by categorizing an important prognostic variable (Buettner et al., 1997). There are multiple methods to achieve cut point such as sample quantile like median, minimum p-value method (Heinzl and Tempfer, 2001), and change-point models (Wang et al., 2007). Some studies determined cut points by change-point models. Lopez Abente et al., (2010) conducted a study to update changes in CRC mortality incidence. Applying transition change-point model, they concluded that, compared to the past, CRC incidence increased considerably, while CRC incidence and mortality trend has experienced opposite patterns (López-Abente et al., 2010). Contal et al., (1999) used change-point method in semi parametric Cox model to determine cut point in breast cancer diagnosis age. Cut point of age was obtained 41 for breast cancer diagnosis age in this method which used non-linear effect of age on cancer survival time (Contal and O’Quigley, 1999). 

Change point in age is the age when a process starts to change (Assareh and Mengersen, 2012). There is strong evidence indicating that CRC is preventable and screening according to clinical and practical guidelines is recommended. Knowing the cut point in age is important (Goodman et al., 2006). Therefore, in order to obtain a cut point in disease diagnosis age, a change-point model is utilized to estimate cut point. The advantage of this model is the consideration of immune patients. In other words, if the cancer is diagnosed in early stages, a proportion of patients is likely to have longer survival time than others and might even cure. In standard survival models such as Cox Proportional Hazards and parametric models, it is assumed that all subjects finally experience the event. Using these models are not suitable in cases that there are cured people or people with long survival time (Farewell,1982; Maller and Zhou, 1996). In these cases cure models are used. Survival cure models are utilized to analyze data when there is a chance of survival long time and cured (Machin et al., 2006; Taweab et al., 2015). Survival cure model is utilized in this study to determine cut point in CRC diagnosis age so that exponential mixture cure model with one cut point in age covariate is utilized (Othus et al., 2012). We obtained cut point in CRC diagnosis age and cure rate in CRC patients in Tehran, Iran. In this model, likelihood function is divided into two sections: the first section is associated with data smaller than change point parameter and the second is related to data larger than change point parameter. Then model parameters are estimated by numerical search methods. Determining cut point in cancer diagnosis age improves treatment planning. Therefore this paper aims to obtain cut point in CRC diagnosis age. 

## Material and Methods

In this longitudinal study, the data of CRC patients who were registered in Taleghani hospital in Tehran, Iran were utilized. Cancer was diagnosed between 1985 and 2012 and these patients were followed until 2013. In order to reach the information, the telephone numbers were recorded. Some demographic characteristics such as diagnosis age of CRC and the last condition (death as a result of CRC, etc.) were recorded. Survival time was considered from entry to the study to death as a result of CRC or CRC return. Of 475 patients, 345 entered the study and other removed because of lack of information. Mixture survival cure model was fitted to the data due to the presence of immune individuals. The data were analyzed by R version 2.15.0 and SPSS version 20. The goodness of fit of cure model was investigated and then likelihood function of exponential cure model along with change point in age covariate was written and maximum likelihood estimation of the model parameters were obtained using numerical search methods.

*Mixture cure model and change point mixture cure model*

Two types of cure models are mixture and non-mixture (Li et al., 2007; Taweab et al., 2015). In mixture cure model, patients fall into two groups: cured and susceptible. In each mixture cure rate model there are two groups of people: cured and susceptible. Survival function for mixture cure model is written as follows that in it p is cure rate.

where SU(t) is susceptible or non-cured survival function. Some distributions such as Lognormal and Weibull are utilized for modeling in (1) (Maller and Zhou, 1996; Li et al., 2007).

For this model all people are alive at the beginning of the study (S(0)=1) and a proportion of people (p) remain alive at the end of the study (S(∞)=p). (Machin et al., 2006). The main objective in mixture cure models is to estimate cure ratio. (Maller and Zhou, 1996; Corbiere et al., 2009). 

Before using cure models, 2 assumptions need to test: the presence of cured fraction and long enough duration of follow-up.

In some cases, long enough follow-up duration can be probed from clinical experiences. In cure models, parametric and non-parametric tests are used to study the cured patients in data (Maller and Zhou, 1996).

In change point exponential cure model, likelihood function is divided into two sections: less than and greater than the change point parameter (Othus et al., 2012). In each section, exponential distribution was used. After writing likelihood function, we can estimate hazards in two parts and change point in the age. Likelihood function of change point exponential cure model is:

Where in it f(t): density function of event time variable, S(t): survival function of event time variable, τ: change-point parameter for X quantitative variable and δ is event condition for each individual (0 for censorship and 1 for event). After smoothing of this function, parameters (change point parameter and hazards in two parts) are estimated by maximum likelihood method and numerical search methods.

## Results

A total of 345 patients entered the study. The mean age was 56.51 ±11.52. Of this number, 165 patients (47.8%) were female and 180 (52.2%) were male. Of 345 patients who entered the study, the survival time of 93 patients (27%) was accurately recorded and 255 patients (73%) were considered right censoring (there was not accurate survival time for them).

**Table 1:** Estimation of Parameters and Parameter Intervals for Colorectal Cancer in Tehran

Parameter	Estimate	95% CI
Change-point estimate ( τ̂)	50.46	(46.50,54.42)
Cure estimate for age < τ̂	0.65	(0.45,0.85)
Hazard estimate for age < τ̂	0.16	(0.02,0.30)
Cure estimate for age ≥ τ̂	0.31	(0.01,0.68)
Hazard estimate for age ≥ τ̂	0.13	(0.03,0.23)

**Figure 1:**
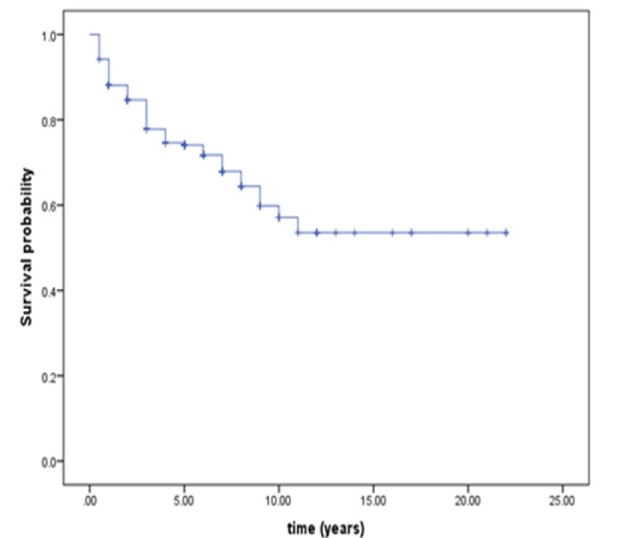
Estimated Kaplan-Meier Graph for Colorectal Cancer Patients in Tehran

**Figure 2:**
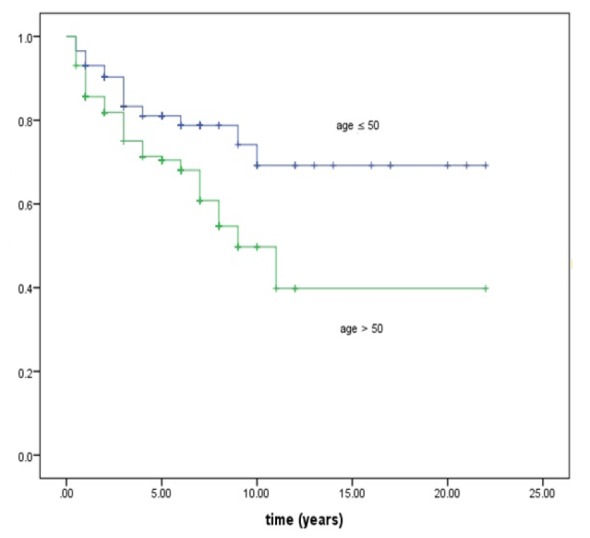
Estimated Kaplan-Meier Graph by Age for Colorectal Cancer Patients in Tehran

The first assumption that should be tested is sufficient follow-up. Figure 1 shows Kaplan-Meier graph for 345 subjects. From Figure 1 it is clear 25-year sufficient follow-up time. Kaplan-Meier graph becomes flat after 10 years and remains fixed until the end of follow-up. Also the second assumption is the presence of cured people in the study. It is clear from Figure 1, high percentage (50%) of patients is not likely to die, and they have lived 10 years after beginning of the study until the end of the study. Also parametric test confirmed it. Therefore, goodness of fit of mixture cure model was appropriate.

In order to estimate cut point in diagnosis age, change point exponential cure model was fitted to the data. Table 1 shows the results of model. The results of table 1 show that cut point in age of colorectal cancer is nearly 50 years. Also, cure ratio is 50% among the patients who are at or younger than 50 (younger individuals) and cure ratio is 31% among the patients who are older than 50 (older individuals). Hazard rate are 0.16 and 0.13 among young and old patients, respectively.

Since cut point in the age was obtained 50, Kaplan-Meier graph were drawn for two groups of patients: younger-than 50 and older-than-50 (Figure 2). Survival time of the older is more than that of the younger; however, the graphs are close to each other in the first 3 years. Kaplan-Meier graph becomes flat after 10 years among the younger group, and almost 10.5 years among the older group. It seems that change-point model is an appropriate model. 

## Discussion

In this study a cut point for the age covariate in CRC cancer was determined. It was nearly 50 years. Fu et al., (2014) studied on appropriate cut point and the impact of age on CRC survival in China. In this study, pre-defined age groups were formed. Then, with comparison survival graphs in pre-defined age groups, they concluded that cut point of 35 years is appropriate for CRC. Our result is far different from Fu et al., (2014) study. This difference is associated with different statistical population and statistical methods. In some studies, Cut point 50 was utilized which is consistent with this study (Woods et al., 2010; Safaee et al., 2011; Lieu et al., 2014; Win et al., 2014). In some studies, many cut points were utilized for age (Dominitz et al., 1998; McMillan et al., 2003).

In this study, the older-than-50 was more than the younger by 36%. In other words, the disease mostly occurs in old age. Some research in Iran shows that cancer is diagnosed after 50 in more than 50% of cases (57.2%). They are the target in screening programs (Moghimi et al., 2008). On the contrary, Lutgens et al. study showed that percentage of younger-than-50 group is more which is inconsistent with this study (Lutgens et al., 2013). Cure ratio among younger age (50 and younger) was obtained 65% which is higher by 34% compared to older ages (older than 50). In other words, if cancer occurs in younger ages, a higher proportion of people would recover. CRC patients who are 50 or younger yield longer survival time than those who are older than 50 at the time of cancer diagnosis. Fu et al., (2013) compared survival in two groups: older than 30 and younger than 30. They concluded that younger patients have shorter survival time than older ones, which is inconsistent with this study. This contradiction might be associated with different cut point and population. 

The results of some other studies also show that as age rises, hazard increases, and survival decreases which is consistent with this study (Laurent et al., 2003; McMillan et al., 2003; Samowitz et al., 2005; Majek et al., 2012; Dominitz et al., 1998).

In this study, the mean age was reported 56, which is consistent with the result of some other studies which reported the age between 40 and 50 (Win et al., 2014; Morikawa et al., 2013; Karimi et al., 2013; Woods et al., 2010). Some studies reported 40-50 as mean age (Safaee et al., 2011) and some others reported older than 60, which is inconsistent with this study. 

In this study, we obtained cut point in the age using a cure model with change point in age covariate. Consideration of the immune patients in the study using cure model, estimation of age change point using a parametric model, and simultaneous estimation of cure and exponential distribution rate in a model for both sections (data before and after the age change point) is advantages of this model. The results show that family history of cancer is an important CRC occurrence (Garland and Garland, 1980; Abadi et al., 2013). It is also one limitation due to lack of exact record of cancer family history. Applying other parametric models such as Weibull and Lognormal, which have more flexibility, is recommended. 

To determine a cut point in multistage cancer diagnosis age, a cure model with change point in a quantitative variable can be utilized. In this study, using mixture cure model along with change point in age, we obtained cut point in diagnosis age, hazard ratio, and percentage of immune patients prior and after the diagnosis age. Determine a cut point in diagnosis age and the analysis of prognostic groups are highly regarded in screening planning. 
